# Are psychologists aware of group psychological abuse? A survey on awareness of the phenomenon among Italian psychologists

**DOI:** 10.3389/fpsyg.2026.1749074

**Published:** 2026-03-26

**Authors:** Lorita Tinelli, Paride Destratis, Antonietta Curci, Alexa Schincariol, Giulia Melis, Cristina Scarpazza

**Affiliations:** 1 Ce.S.A.P. Centre for the Study of Psychological Abuse, Bari, Italy; 2 Department of Education, Psychology, Communication, University of Bari “Aldo Moro”, Bari, Italy; 3 Padova Neuroscience Center (PNC), University of Padova, Padova, Italy; 4 Department of General Psychology, University of Padova, Padova, Italy; 5 Department of Biomedical Sciences, University of Padova, Padova, Italy; 6 IRCCS San Camillo Hospital, Venezia, Italy

**Keywords:** awareness among psychologists, cults, group psychological abuse, psychological manipulation, undue influence

## Abstract

**Introduction:**

Psychologically abusive groups are characterized by systematic influence, relational control, and leadership structures that foster dependency; however, empirical data on how mental health professionals conceptualize these phenomena remain limited. The present study investigated Italian psychologists’ knowledge, representations, and professional experiences regarding psychologically abusive groups.

**Methods:**

An online survey was administered to 232 psychologists across Italy between November 2024 and April 2025. The questionnaire explored self-assessed knowledge, information sources, representations of groups, members, and leaders, perceived abusive strategies, clinical experiences, diagnostic formulations, and training needs.

**Results:**

Limited formal preparation, with many participants relying primarily on non-academic sources despite rating scientific materials as more reliable. Abusive groups were predominantly described in terms of manipulation, isolation, and psychological control, while leaders were characterized by charisma, narcissistic grandiosity, and exploitative tendencies. In contrast, members were mainly portrayed as psychologically vulnerable, suggestible, and in need of belonging. Nearly half of respondents reported direct clinical contact with individuals involved in such groups. Reported clinical profiles were heterogeneous, with recurrent internalizing symptoms, trauma-related presentations, and dependency-related features.

**Discussion:**

Overall, findings reveal a partial alignment with contemporary systemic models of group psychological abuse, alongside a persistent tendency to frame involvement in individual vulnerability terms. The study underscores the need for clearer conceptual frameworks, evidence-informed terminology, validated assessment tools, and structured professional training to support clinical and forensic practice in this area.

## 1 Introduction

The phenomenon of groups with sectarian characteristics represents an area of considerable psychosocial and criminological interest, characterized by marked conceptual ambiguity and enduring theoretical and empirical controversies (Robbins and Zablocki, 2001). According to classical definitions, a *sect* is a religious movement that separates from a preexisting religion, partially modifying its doctrines and practices (Stark and Glock, 1965). However, more recent researchers suggest that these groups need not necessarily be rooted in religious themes; rather, they may be founded upon broader belief systems or ideals, often corresponding to what (Taylor, 2006) terms *ethereal ideas*: abstract and vague concepts open to multiple and sometimes contradictory interpretations. From this perspective, Hassan (2015) identifies four main types of abusive groups based on their central focus: religious (grounded in spiritual doctrines or the leader’s revelations), political (centered on ideological allegiance), psychotherapeutic or educational (promising personal growth or “enlightenment” through often costly and isolating programs), and commercial (organized around profit-oriented ideologies). It is important to note that these categories are not mutually exclusive: commercial dynamics may also characterize other types of abusive groups–particularly psychotherapeutic, educational, or self-improvement organizations–highlighting the fluid and overlapping nature of abusive group practices. The notion of *sect* therefore extends beyond the purely religious or ideological domain, encompassing any organization that systematically employs manipulative and coercive strategies exceeding ordinary processes of social influence (Rodríguez-Carballeira et al., 2015; Singer, 1996).

In recent years, research has progressively shifted its focus from the religious or ideological dimensions of these groups to the abusive practices that define their functioning (Dubrow-Marshall and Dubrow-Marshall, 2016). Within this framework, the construct of Group Psychological Abuse (GPA) has emerged, defined as a systematic process in which leaders and other group members repeatedly perpetrate abusive strategies to dominate individuals within a group, restructure their identity, and ultimately maintain dependence (Almendros et al., 2011; Rodríguez-Carballeira et al., 2015). These strategies (intentional, planned, and pervasive) seek to achieve comprehensive control over members, subordinating their well-being to the interests of the leader or the organization (Almendros et al., 2011; Langone and Chambers, 1991; Singer and Lalich, 1995).

### 1.1 Characteristics of abusive groups and strategies

Group Psychological Abuse contexts are characterized by totalitarian and rigidly controlled environments, where strict conformity to specific behavioral, cognitive, emotional, and social expectations imposed by the leadership are enforced (Rodríguez-Carballeira et al., 2015). Such organizations often exhibit a hierarchical and pyramidal structure, with power concentrated in the hands of a few individuals or a charismatic leader who, through mechanisms of manipulation and isolation, fosters a sense of dependency that serves to maintain control over the group (Coates, 2012; Singer, 2003; Zhang, 2023).

The literature has identified three fundamental characteristics of GPA contexts (Rodríguez-Carballeira et al., 2015): (i) the abusive and manipulative nature of the strategies employed to retain members; (ii) their prolonged and repetitive application over time; and (iii) the subjugation and domination of members as the ultimate purpose of such strategies. Affiliation with the group typically follow a progressive sequence of phases: recruitment, integration, and maintenance, and encompass a structured set of intentional strategies that form the core of undue influence processes (Rodríguez-Carballeira et al., 2015): (i) isolation; (ii) control and manipulation of information; (iii) control over personal life; (iv) emotional abuse; (v) indoctrination into an absolute belief system; (vi) imposition of a unique and extraordinary authority.

Psychological manipulation should not be understood as a “magical” or irrational phenomenon, but rather as a sophisticated persuasive process that exploits well-established principles of social psychology (Di Nunno et al., 2023). Among these, mechanisms of self-selection and conformity play a particularly significant role: individuals who express doubts tend to leave the group in its early stages, fostering the development of an increasingly homogeneous and conformist environment. Adhesion to the group typically unfolds gradually. Progressively escalating demands give rise to a kind of *procrastination of disobedience*, where the immediate psychological cost of resisting seems greater than that of incremental submission–a process reminiscent of Milgram’s (1963) and Asch (2016).

Within this process, persuasion is consolidated through mechanisms of constrained choice and framing, which reshape individuals’ perception of events and their value hierarchies, rendering otherwise irrational decisions coherent and meaningful. This progression culminates in a state of *bounded choice* (Lalich, 2000), in which even extreme actions, such as absolute devotion or substantial financial donations, are experienced as autonomous and consistent, despite originating from a gradual and highly refined process of psychological conditioning.

### 1.2 The charismatic leader

Charismatic leadership is a recurring feature in both theoretical and empirical descriptions of abusive groups (Dayan, 2018; Dole, 2006; Freckelton, 1998; Galanter, 1983; Lifton, 1961; Robbins, 2002). It is grounded in a perception of exceptionality and omnipotence, often imbued with transcendent or quasi-divine qualities. Within this framework, the *cult of personality* assumes a structural function: the leader becomes the primary affective, moral, and cognitive reference point for the group. From a psychosocial perspective, charismatic authority is sustained through the continuous recognition granted by followers, upon which the legitimacy of power ultimately depends (Brodie, 2010; Emerson, 1962). In this sense, the “implantation of belief in the leader’s special qualities” constitutes a powerful form of GPA, as it underpins all subsequent strategies of control and manipulation.

The figure of the charismatic leader therefore represents the central element of most psychologically abusive groups. Their authority tends to consolidate progressively, eventually becoming an object of veneration among followers. This figure centralizes decision-making power, demands total delegation, and legitimizes their position through the alleged possession of special powers or knowledge, which serve to justify various forms of psychological, economic, and sometimes sexual exploitation (Lifton, 1961; Singer, 1996).

A substantial body of research has shown that such leaders often exhibit traits associated with antisocial and narcissistic personality disorders (Pompili et al., 2023), characterized by manipulation, aggressiveness, disregard for the rights of others, grandiosity, and an excessive need for admiration. These traits are frequently accompanied by anger, hypersensitivity to criticism, and vindictive behavior (Burke, 2006; Oakes, 2015; Rahmani et al., 2019; Shaw, 2014). In certain cases, paranoid features may also emerge, such as distrust and suspicion (Goldberg, 2012).

Leaders vary widely in background, ideology, and behavioral and motivational characteristics, resulting in distinct profiles influenced by the sociocultural context and the specific nature of the group they lead. Despite these contextual and ideological differences, the literature consistently identifies several core components that characterize leaders of abusive groups. Singer and Lalich (1995) highlight three fundamental dimensions: self-proclamation and persuasive capacity, through which the leader presents themselves as the custodian of special truths; charisma and authority, which facilitate members’ submission; and the centralization of collective veneration, often accompanied by demands for personal sacrifice and the severing of family ties. According to Taylor (2006), charismatic power rests on two interdependent dimensions: *charisma*, which enables the leader to attract and captivate followers, and *the motivation for control*, which drives them to maintain influence through manipulation and coercive strategies.

### 1.3 Who joins (and why they stay): characteristics of members?

Joining an abusive group is not an isolated event, but rather a gradual process consisting of a sequence of distinct decisions, each of which progressively reduces the psychological distance from the group’s authority (Di Nunno et al., 2023; Galanter, 1983). Individuals, often without full awareness of this progression, take a series of small steps, such as attending an introductory meeting, followed by a more intensive seminar or a residential period, which consolidate commitment and facilitate the internalization of group norms.

Contrary to common representations, empirical evidence does not support the notion that all individuals who join abusive groups display preexisting psychopathology (Aronoff et al., 2000). Rather, adherence appears to arise from the interaction between situational vulnerabilities–such as transitional life phases, personal or existential crises, conflicted family relationships, lack of social support networks, tendencies toward emotional dependency, and unmet needs for belonging and meaning–and an initial relational and identity-based offer that is strongly reinforcing, often characterized by warmth, idealism, and a shared sense of mission (Aronoff et al., 2000; Levine and Salter, 1976; Spero, 1982). More broadly, research on coercive control and relational abuse conceptualizes vulnerability as contextually and relationally constructed, arising from asymmetric power dynamics, progressive restriction of autonomy, and emotional dependency rather than from fixed personality profiles (Herman, 2015; Stark, 2007). Within such frameworks, susceptibility is understood as the outcome of structured influence processes operating within periods of psychological or social transition. While certain interpersonal orientations–such as high trust or affiliative motivation–may shape social engagement, these reflect normative prosocial traits rather than pathological weakness. Contemporary personality research distinguishes between prosocial “light” traits and antagonistic “dark” traits (Kaufman et al., 2019), suggesting that benevolent interpersonal dispositions may be asymmetrically exploited in environments structured around manipulation and control.

A useful interpretative framework is the analogy with addiction disorders, stemming from a theoretical line that compares cult involvement to substance use disorders (Booth, 1991; Roy, 1998). Although the analogy is conceptual and the mechanisms underlying group psychological abuse differ from clinical addiction, some behavioral dynamics observed in high-control environments – including progressive escalation of commitment, persistence despite negative consequences, salience attribution, and difficulty disengaging – resemble processes described in contemporary models of addiction (e.g., incentive sensitization and compulsive engagement; Koob and Volkow, 2010; Robinson and Berridge, 2008; Volkow et al., 2016). Group commitment indeed exhibits features of persistence despite negative consequences, an initial sense of relief and belonging, and the exclusive centrality of the “harmful but craved” object (group or leader) in thoughts and behaviors (Rousselet et al., 2017).

Within this multidimensional model, continued membership results from the interaction between individual vulnerabilities, the structural characteristics of the group (degree of control, intensity of social reinforcement, ideological rigidity), and the surrounding environment. Conversely, protective factors–such as maintained family ties, the support of significant others, access to therapeutic interventions, or external social networks–constitute crucial variables in processes of disengagement and recovery (Rousselet et al., 2017).

### 1.4 Former members: outcomes and needs

Former members of abusive groups are commonly regarded as victims of prolonged psychological abuse exerted by the leader and other group members. Continuous exposure to such environments is recognized as potentially harmful to mental health, and numerous studies have documented the presence of post-involvement psychological consequences, often complex and multifaceted (Aronoff et al., 2000; Martin et al., 1992). During their time within the group, members may appear socially well-adjusted, sustained by strong conformist pressure and a pervasive demand for social desirability. However, upon disaffiliation, many former members report clinically significant symptoms, including mood and anxiety disorders, relational difficulties, dissociative phenomena, self-destructive tendencies, and, in a substantial proportion of cases, post-traumatic symptoms (Aronoff et al., 2000; Göransson and Holmqvist, 2018; Herman, 2015; Raine and Kent, 2019; Rosen, 2014; Saldaña et al., 2018).

One of the most significant consequences of abusive groups involvement concerns the weakening of reflective and decision-making functions, with a resulting impairment of critical thinking and personal autonomy. Under such conditions, a *pseudo-identity* may develop–constructed and reinforced in response to group pressures–which tends to overlap with the “true Self,” generating identity confusion and decisional ambivalence (Ash, 1985). In some cases, this fragmentation of the Self may manifest through symptoms consistent with an *Other Specified Dissociative Disorder* (Adams, 2008; American Psychiatric Association, 2022; Hassan et al., 2022; Hassan and Shah, 2019; Mesrobian, 2016).

Recent qualitative research has helped to elucidate the ambivalent nature of post-group experiences. Studies conducted with individuals who have exited high-cost religious groups show that disaffiliation often involves, alongside emotions such as fear, shame, guilt, and grief for the loss of previous relationships and identity, positive experiences of relief, freedom, joy, and empowerment, associated with the process of reconstructing the Self and rebuilding social networks (Björkmark et al., 2022).

The exit from an abusive group therefore entails a complex identity renegotiation and a redefinition of belonging ties–an experience many former members describe as “living between two worlds” (Björkmark et al., 2022). This transition may be accompanied by high levels of isolation, economic instability, and stigmatization. Factors such as social and familial support, as well as the internal disillusionment arising from recognizing the contradictions of the group or its leader, appear to play a decisive role both in the decision to leave and in the subsequent reconstruction of personal autonomy. For these reasons, psychological and social interventions for former members should consider their specific experiential context and the distinctive dynamics of dependency and identity reconstruction that characterize these trajectories.

Effective interventions should therefore integrate clinical and psychosocial perspectives, promoting, on one hand, the recovery of autonomy and self-trust, and on the other, the rebuilding of external support networks. In this regard, the involvement of family members and practitioners specifically trained in the dynamics of GPA phenomena constitutes a crucial protective factor for fostering resilience and preventing relapses or the emergence of new forms of dependency (Björkmark et al., 2022; Rousselet et al., 2017).

It is therefore unsurprising that many former members seek help from mental health professionals (Saldaña et al., 2018). Similarly, requests for support also come from the relatives of individuals who remain within such groups, who, while attempting to “recover” their loved ones, often experience significant psychological distress themselves.

Although academic research has increasingly contributed to elucidating the complexity of the phenomenon (e.g., Rodríguez-Carballeira et al., 2015; Saldaña et al., 2018), myths and distorted beliefs about abusive groups and their members persist in the general population (Robbins and Zablocki, 2001). Such misconceptions suggest that even among mental health professionals, knowledge of the phenomenon may remain uneven or incomplete. Regrettably, no studies so far have examined psychologists’ awareness of GPA in Italy or in other national contexts. The absence of empirical benchmarks on professional awareness therefore justifies the exploratory nature of the present study. This knowledge gap is also exacerbated by the lack of reliable epidemiological data on the GPA phenomenon itself: the most recent systematic estimate, published nearly 20 years ago, indicates that more than 2.5 million individuals in North America have been involved in abusive groups (McCabe et al., 2007). To our knowledge, no more recent prevalence studies have been conducted, and no epidemiological data are available for Europe or Italy. The persistent absence of empirical monitoring–likely linked to the secrecy, stigma, and limited institutional reporting surrounding these groups–underscores the need to investigate psychologists’ awareness of group psychological abuse.

### 1.5 Aims of the study

In light of these considerations, the primary aim of the present study was to investigate the level of knowledge, awareness, and professional representations of group psychological abuse among psychologists in Italy. Specifically, the objective was to assess psychologists’ self-perceived knowledge of the topic, the sources from which they acquired such information, and their evaluation of its overall reliability and quality. The study also aimed to explore the spontaneous conceptualizations of participants regarding the psychological profiles of group members and leaders, the strategies of coercion and manipulation typically employed within such contexts, and the psychological consequences commonly experienced by former members. Additionally, the research investigated the frequency and nature of direct professional encounters with cases involving group-related issues, as well as the diagnostic categories most frequently associated with such cases. Overall, this study was designed to provide an initial nationwide overview of Italian psychologists’ understanding of abusive groups dynamics and to lay the groundwork for the development of evidence-based training initiatives and clinical assessment tools in this underexplored field.

## 2 Materials and methods

### 2.1 Procedure

Data were collected through an *ad hoc* online questionnaire. The survey was disseminated using a multi-channel recruitment strategy, including institutional mailing lists (e.g., the National Council of Psychologists and regional professional boards), professional and private organizations, and social media platforms. According to data provided by the National Council of Psychologists, more than 145,000 psychologists were registered in Italy in 2025; the dissemination channels used potentially reached approximately 1000 professionals.

Participation was voluntary and anonymous. Before accessing the questionnaire, participants were provided with an online information sheet describing the aims of the study, data protection procedures, and participants’ rights, and were required to provide electronic informed consent. The questionnaire required approximately 12 min to complete. No financial or material incentives were offered. The study protocol was approved by the Ethics Committee of the University of Padua (Protocol Code 1479-b) and was conducted in accordance with the Declaration of Helsinki.

### 2.2 Participants

Participants were psychologists recruited across Italy. Inclusion criteria were: (a) being 18 years of age or older; (b) holding a university degree in Psychology (bachelor’s or master’s level); and (c) providing informed consent to participate in the study and for the use of their data. No minimum number of years of professional experience was required for inclusion.

### 2.3 Questionnaire

The administered questionnaire was divided into several sections, which are presented and described below. The full questionnaire, including all items and response options, is available in the Supplementary materials.

Participants first provided basic sociodemographic and professional information (Section A – Sociodemographic and Professional Profile), followed by items assessing their perceived knowledge and sources of information regarding abusive groups. Respondents were asked to rate their overall level of self-assessed knowledge on the topic, followed by an evaluation of the sources through which such knowledge had been acquired (Sections B1–B2–B3 – Self-assessed Knowledge, Sources of Information, and Overall Perceived Quality of Information). For each source, participants indicated both the amount of information obtained and its perceived quality, allowing the distinction between exposure to information and trust in that information.

Participants were then invited to articulate their conceptualizations of abusive groups. This was first explored through an open-ended prompt asking respondents to describe what they considered the three main defining characteristics of such groups, thus capturing spontaneous representations (Section B4 – Descriptions of Abusive Groups). Subsequently, a series of evaluative statements assessed the extent to which different definitional labels were perceived as applicable to abusive groups, ranging from highly negative framings (e.g., manipulative or violent groups) to more neutral or alternative descriptions (Section B5 – Descriptions of Abusive Groups).

A similar mixed-method approach was adopted to examine representations of individuals involved in abusive groups. Participants were asked to provide an open-ended description of the main characteristics they attributed to group members (Section B6 – Descriptions of Members), followed by a set of Likert-scale items assessing agreement with commonly cited individual traits, such as emotional vulnerability, susceptibility to influence, or prior psychological difficulties (Section B7 – Descriptions of Members).

Leadership within abusive groups was explored through an additional open-ended question, in which participants described the characteristics they considered most typical of group leaders, allowing for the emergence of spontaneous leadership profiles (Section B8 – Descriptions of Leaders).

The questionnaire then shifted to perceived group dynamics and practices. Participants were first asked to list strategies they believed were commonly employed by abusive groups (Section B9 – Group Strategies, Behaviors and Perceived Harmfulness). These qualitative responses were complemented by structured evaluations of specific behaviors, assessed along two parallel dimensions: how typical such behaviors were perceived to be of abusive groups and how harmful they were considered for members (Section B10 – Group Strategies, Behaviors and Perceived Harmfulness).

Further items addressed the consequences of involvement in abusive groups, focusing specifically on former members (Section B11 – Difficulties Among Former Members). Participants rated the extent to which a range of psychosocial difficulties were perceived as common among individuals who had exited such groups (B11.01), as well as the degree to which these difficulties were considered persistent or pervasive over time (B11.02).

Professional experience with abusive groups-related issues was explored in the subsequent part of the questionnaire. Participants reported whether they had encountered cult-related problems in their professional practice, the contexts in which such cases occurred, and whether they had personally intervened in these situations. An open-ended question allowed respondents to indicate the diagnoses most frequently associated with these cases in their experience (Section C1 – Direct Professional Experiences).

The final sections examined participants’ views on terminology, diagnostic practices, and professional training. Respondents evaluated the appropriateness of commonly used expressions describing abusive mechanisms and rated the acceptability of psychological interventions in cases where group members do not explicitly seek help. They were also asked about their awareness of specific psychometric tools used in this field (Sections D1–D2–D3 – Appropriateness of Terminology and Diagnostic Instruments). The questionnaire concluded with items assessing perceived training needs and interest in receiving feedback on the study results (Sections D4–D5 – Training Interest and Feedback Preferences).

### 2.4 Data analysis

Descriptive statistics (means, standard deviations, frequencies, and percentages) were computed to summarize sociodemographic characteristics, professional background, self-assessed knowledge, information sources, and evaluative ratings. For Likert-scale items, mean scores and standard deviations were calculated for each item. Open-ended responses were analyzed using quantitative content analysis based on word-occurrence frequencies, following a semantic-literal coding criterion. Responses were initially reviewed to identify recurring lexical patterns and thematic clusters. These were subsequently organized into mutually exclusive semantic categories, which were further grouped into higher-order conceptual domains. Overly generic or ambiguous expressions that could not be clearly assigned to a specific category were also excluded. This procedure was carried out independently by two researchers. In cases of disagreement, a third researcher replicated the analysis.

Given the exploratory nature of the study, analyses were primarily descriptive. No *a priori* hypotheses were tested, and no inferential statistical comparisons were planned.

## 3 Results

### 3.1 Participants

**FIGURE 1:**
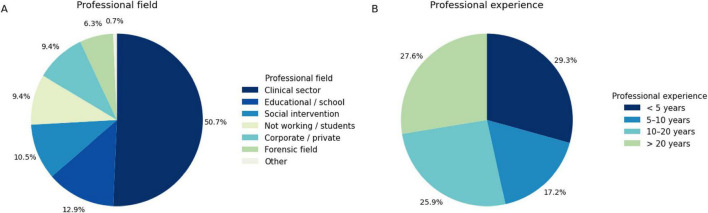
Distribution of participants’ professional background and years of professional experience. **(A)** Shows the distribution of professional fields, with the majority of participants working in the clinical sector. **(B)** Illustrates the distribution of years of professional practice. Percentages refer to the proportion of participants within each category.

A total of 232 individuals holding a degree in psychology completed the survey. The mean age of participants was 44.65 years (SD = 13.68). Regarding educational and professional background, nearly half of the respondents (*n* = 115, 49.6%) indicated that they have completed a specialization in psychotherapy, while 75 (32.3%) held a specialist or master’s degree, 37 (15.9%) a 3-years bachelor’s degree, and 5 (2.2%) a doctoral degree. Of these, 177 respondents (76.3%) were registered members of the professional board of psychologists, while 55 (23.7%) were not registered. The sample was predominantly composed of professionals working in clinical settings, followed by educational and social intervention contexts (Figure 1A). Professional experience was well distributed across career stages, with a substantial proportion of participants reporting more than 10 years of practice (Figure 1B).

**FIGURE 2:**
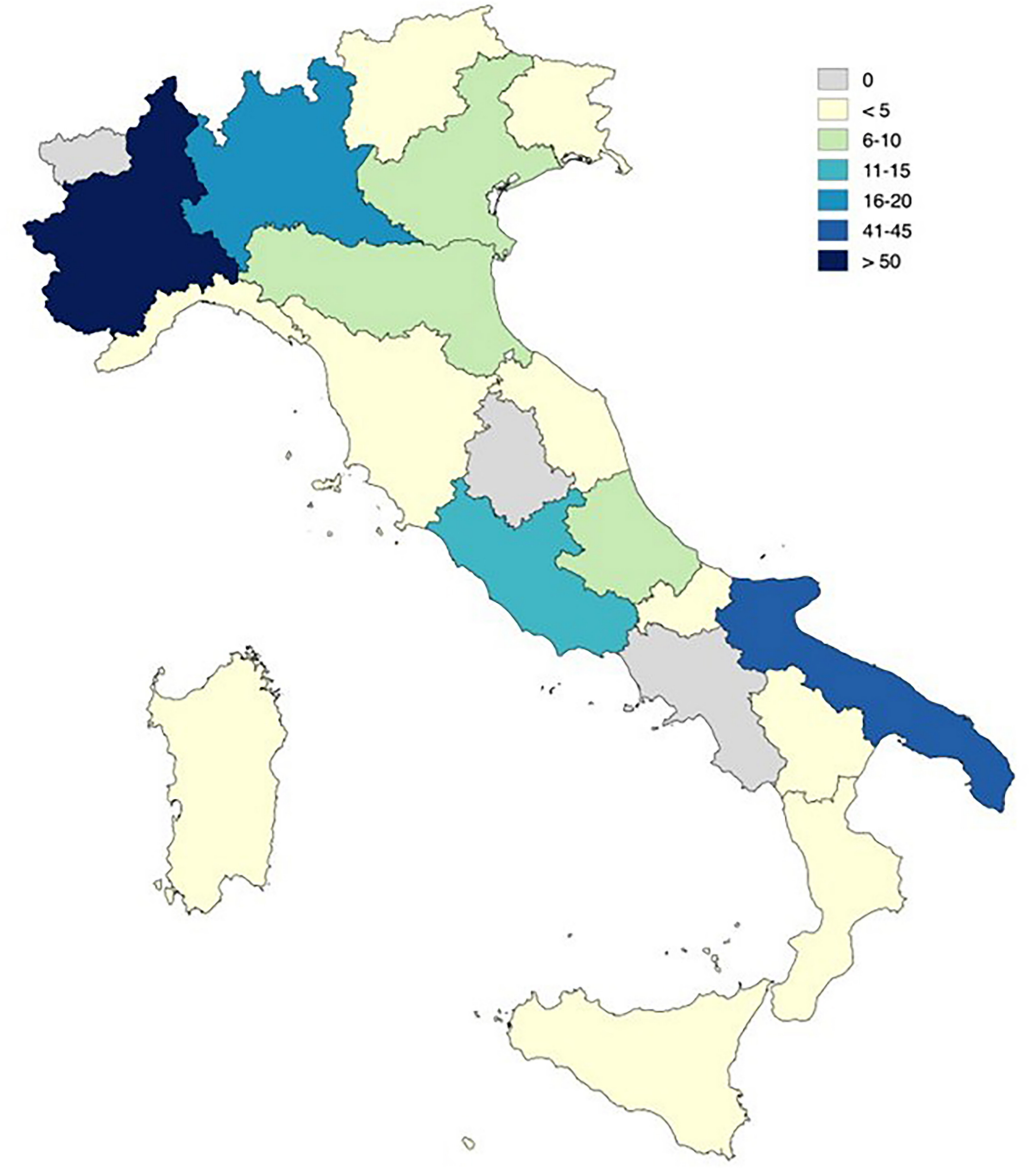
Geographical distribution of Italian professionals who completed the survey. Survey participants are distributed geographically across Italian regions as follows. Piedmont, *n* = 66; Apulia, *n* = 41; Lombardy, *n* = 19; Lazio, *n* = 11; Abruzzo, *n* = 6; Veneto, *n* = 6; Emilia-Romagna, *n* = 5; Friuli-Venezia Giulia, *n* = 3; Sardegna, *n* = 3; Sicily, *n* = 3; Toscana, *n* = 3; Basilicata, *n* = 2; Liguria, *n* = 2; Marche, *n* = 2; Trentino-Alto Adige, *n* = 2; Calabra, *n* = 1; Molise, *n* = 1.

Geographically, the sample was predominantly drawn from northern and southern Italy. The largest proportion of respondents resided in Piedmont (37.5%), followed by Puglia (*n* = 41) and Lombardy (*n* = 19). See Figure 2 for a geographical distribution of the survey respondents.

### 3.2 Self-assessed knowledge of GPA phenomenon

Frequency analyses conducted on Section B1 revealed that half of the respondents reported having little or no knowledge of the phenomenon (50%), whereas only 4% indicated possessing extensive knowledge. Additionally, 27% reported having sufficient knowledge, and 19% reported having fairly good knowledge. At the end of the survey, 98% of participants (Section D4) considered it important to implement training initiatives on this topic, and 89% (Section D5) expressed interest in receiving the results of the present study.

### 3.3 Sources of information and perceived quality

The analysis of information sources is summarized in Table 1, which reports both the frequency of use and the perceived quality of each source. Overall, the use of information sources showed a heterogeneous distribution. The Internet emerged as the most frequently used source, followed by newspapers and television, scientific literature, and popular magazines. In contrast, training courses and seminars were the least frequently reported sources of information. Despite their limited use, training courses and seminars received the highest quality ratings, suggesting that although fewer participants relied on these sources, those who did perceived them as particularly informative. Similarly, personal experience, clients or patients, and professional colleagues–moderately used sources–were associated with relatively high perceived quality. By contrast, newspapers and television, as well as internet sources, despite being among the most frequently used, were rated as lower in quality compared to more specialized or experience-based sources.

**TABLE 1:** Use and perceived quality of information sources.

Information source	Use Mean (SD)	Quality Mean (SD)*
Internet	2.96 (1.15)	2.70 (0.93)
Newspapers/television	2.68 (1.00)	2.66 (0.93)
Scientific literature	2.26 (1.21)	2.79 (0.90)
Popular magazines	2.05 (0.93)	2.72 (0.91)
Professional colleagues	1.92 (1.22)	2.84 (0.96)
Clients/patients	1.81 (1.16)	2.81 (0.89)
Personal experience	1.80 (1.24)	2.89 (0.97)
Training courses	1.59 (1.09)	3.05 (0.97)
Seminars	1.59 (1.06)	3.07 (1.00)

*Quality ratings were calculated only among respondents who reported having used the source (i.e., score > 1).

### 3.4 Perceptions of abusive groups

A total of 194 responses to the open-ended question (Section B4) were analyzed, five participants explicitly stated that they did not know how to respond. Overall, abusive strategies, both physical and psychological (*manipulation, coercion, mind control*), emerged as the most frequently cited characteristic of abusive groups. Specifically, 104 respondents (54%) referred to practices involving psychological manipulation and various forms of control. Closely associated with this dimension was isolation (*separation, relational closure, social detachment*), mentioned by 95 participants (49%). The presence of a strong leadership figure (*charismatic leader, guru, authoritarian leader*) was also prominently reported (*n* = 52; 27%). Beyond leadership, several responses emphasized cognitive and structural features of abusive groups. Rigid or dichotomous thinking (*dualism, rigid rules, imposition of norms*) was identified by 27 respondents (14%), while exclusivity (*elitism, exclusive group*) was mentioned by 26 participants (13%). Hierarchical structure (*pyramidal organization, hierarchy, the presence of a leader at the top and subordinates below*) was reported by 20 respondents (10%), pointing to vertically organized power dynamics. Totalizing dynamics (*totalizing affiliation, total life investment in the cult’s beliefs*) were indicated by 17 participants (9%), while secrecy (*hidden activities, restricted access to information*) was mentioned by 14 respondents (7%).

Less frequently, participants referred to dogmatism (*dogmatic thinking, messianic ideology, unquestionable beliefs, fanaticism*) (*n* = 9; 5%) and recruitment practices (*proselytism, love bombing, loyalty-building strategies*) (n = 9; 5%). Identity loss (*depersonalization, deindividuation, submission*) and rituality (*initiation rites, ritual practices*) were each reported by 8 participants (4%). Finally, followers’ vulnerability (*fragility, suggestibility, need for belonging, dependency*) was mentioned by six respondents (3%).

In Section B5, mean ratings and standard deviations for each label are reported in Table 2. Overall, labels varied in their perceived representativeness and salience, with lower mean ratings associated with more neutral or positive expressions and higher mean ratings associated with more negatively connoted characterizations.

**TABLE 2:** Descriptive statistics (mean and standard deviation) for the perceived representativeness and salience of abusive groups’ labels.

Label	M (SD)
Manipulative and violent groups	3.81 (0.93)
Exploitative groups	3.80 (1.05)
Radical and extremist groups	3.70 (1.00)
Deviant religious groups	3.57 (1.07)
Minorities and divergent groups	2.48 (1.12)
Legitimate alternative groups	1.75 (0.89)

### 3.5 Descriptions of members

A total of 206 responses to the open-ended question (Section B6) were analyzed. Overall, participants tended to describe members of abusive groups primarily in terms of vulnerability. Indeed, 135 respondents (65%) referred to characteristics such as *fragility*, *psychological vulnerability*, *weakness*, or being *victims*. In a similar vein, 63 participants (31%) portrayed abusive group members as *suggestible*, *manipulable*, *easily influenced*, or *credulous*. A substantial proportion also emphasized relational and identity-related aspects. Eighty-seven participants (42%) mentioned the *need for belonging*, *affiliation*, the *need to be in a group*, or traits associated with dependency. These representations were often accompanied by references to *loneliness*, *isolation*, or *marginalization* (*n* = 44; 21%).

Additionally, 39 participants (19%) described members of abusive groups as characterized by *insecurity*, *low self-esteem*, or *low self-efficacy*. Other responses highlighted contextual or existential elements. Twenty-two participants (11%) referred to experiences such as *crisis*, *trauma*, *mourning*, or a *difficult period*, while 27 (13%) mentioned a *search for meaning*, *search for answers*, or a *purpose in life*. A further 20 responses (10%) explicitly described members of abusive groups as being *in need*, seeking *care* or *help*. Finally, 15 participants (7%) attributed membership to *low education*, *limited critical thinking*, or broader *cultural limitations*.

The analysis from Section B7 indicates that participants primarily associated members of abusive groups with psychological and emotional traits, such as *emotional vulnerability* and *suggestibility*. Conversely, factors such as *previous psychological disorders*, *weakness of character*, and *low educational attainment* were considered less relevant. Descriptive statistics are reported in Table 3.

**TABLE 3:** Descriptive statistics (mean and standard deviation) for perceived characteristics of members of abusive groups.

Characteristic	M (SD)
Emotional vulnerability	4.08 (0.93)
Suggestibility	4.03 (0.98)
Weakness of character	3.08 (1.17)
Previous psychological disorders	2.75 (1.02)
Low educational attainment	2.56 (1.17)

### 3.6 Profile of leaders

A total of 200 responses to the open-ended question in Section B8 were analyzed. Overall, participants described group leaders primarily in terms of manipulation. Indeed, 116 respondents (58%) referred to them as *manipulators*, *exploitative*, or *gaslighters*. Alongside this dimension, 111 participants (56%) emphasized traits such as being *charismatic*, *magnetic*, *seductive*, or *fascinating*, and 99 (50%) portrayed them as *narcissistic*, *egocentric*, characterized by *grandiosity* or a *sense of superiority*.

Other responses focused on dominance and control. Thirty-seven participants (19%) described leaders as *authoritarian*, *tyrannical*, *dominant*, or *controlling*. In addition, 34 respondents (17%) highlighted their being *persuasive*, *skilled speakers*, or effective *communicators*, while 33 (17%) explicitly mentioned *psychopathic*, *antisocial*, *personality disorder*, or *paranoid* traits.

Less frequently, participants referred to motivations centered on *power*, *ambition*, or a *desire for control* (*n* = 16; 8%), as well as to *cynical*, *amoral*, *deceitful*, or otherwise *deviant* characteristics (*n* = 15; 8%). Explicit depictions of leaders as *sadistic*, *violent*, *aggressive*, or *ruthless* were reported by 13 participants (7%). Intelligence-related qualities such as being *intelligent*, *cunning*, *clever*, or *shrewd* appeared in 11 responses (6%), whereas references to *messianic* roles, being a *savior*, claiming *exclusive truth*, or expressing a *delusional vision* were comparatively rare (*n* = 6; 3%).

### 3.7 Group strategies and behaviors

A total of 193 responses to the open-ended question in Section B9 were analyzed. Eight participants indicated that they did not know how to respond or provided answers unrelated to the question and were therefore excluded from the analysis.

Overall, participants most frequently described abusive groups as relying on manipulation and persuasion. Ninety-six respondents (50%) referred to *manipulation*, *persuasion*, *promises used to convince individuals to join*, or *gaslighting*. Similarly, 76 participants (39%) highlighted practices aimed at *isolating members*, *cutting off previous ties*, or *excluding them from other social contexts*.

Fifty-seven respondents (30%) described abusive group as using *indoctrination*, *inculcating ideas*, *brainwashing*, *coercion*, or broader forms of *control over thoughts and behaviors*. Forty-six participants (24%) mentioned *love bombing*, referring to *fake love* or an *intense welcoming attitude* as a strategy to attract and retain members.

In addition, 39 participants (20%) emphasized strategies centered on creating *a sense of belonging*, *providing identity*, and strengthening *group cohesion*, while an equal proportion (20%) referred to *threat*, *psychological abuse*, *guilt induction*, *blackmail*, or *fear*. Less frequently, 17 respondents (9%) mentioned *gradual recruitment strategies*, such as *progressive approach* or *proselytism*, and 12 (6%) explicitly referred to *exploitation* as a technique used by abusive groups.

In Section B10, participants rated a series of behaviors according to two dimensions: (i) the extent to which they considered each behavior characteristic of abusive groups (B10.01), and (ii) the extent to which they perceived these behaviors as harmful to members (B10.02). Table 4 presents the mean scores and standard deviations for both dimensions.

**TABLE 4:** Perceived typicality and harmfulness of GPA.

Behavior	Characteristic M (SD)	Harmfulness M (SD)
Requiring full identification with group ideology	4.68 (0.59)	4.55 (0.78)
Idealizing the group and rejecting outsiders	4.66 (0.65)	4.62 (0.69)
Imposing ideology above individuals and laws	4.61 (0.66)	4.58 (0.72)
Granting the leader absolute authority	4.60 (0.63)	4.70 (0.66)
Monitoring members’ behavior	4.58 (0.65)	4.47 (0.79)
Exploiting members’ sense of guilt	4.50 (0.76)	4.75 (0.62)
Demanding total and enthusiastic commitment	4.45 (0.72)	4.26 (0.91)
Encouraging self-sacrifice for the group	4.33 (0.81)	4.62 (0.69)
Controlling members’ activities and leisure time	4.31 (0.85)	4.40 (0.85)
Denigrating critical thinking	4.31 (0.92)	4.69 (0.64)
Strategically granting forgiveness after inducing guilt	4.27 (0.91)	4.64 (0.67)
Regulating emotional and sexual relationships	4.24 (0.91)	4.72 (0.64)
Attacking critics through ad hominem arguments	4.21 (0.91)	4.53 (0.75)
Manipulating language and meanings	4.19 (0.96)	4.52 (0.75)
Controlling members’ finances	4.07 (0.98)	4.64 (0.68)
Weakening members’ physical and psychological condition	4.06 (1.03)	4.77 (0.56)
Negatively reconstructing members’ past and identity	4.05 (1.08)	4.61 (0.68)
Inducing confessions of deviant behaviors or thoughts	4.04 (1.08)	4.53 (0.77)
Distracting members from work, studies, or hobbies	4.01 (0.97)	4.53 (0.81)
Isolating members in group residences	3.91 (1.11)	4.60 (0.74)
Intimidating or threatening members	3.76 (1.10)	4.69 (0.65)
Promoting positive feelings among members	3.76 (1.13)	3.29 (1.29)
Denigrating, humiliating, or rejecting members	2.92 (1.38)	4.46 (0.90)

Mean (M) and standard deviation (SD) of participants’ ratings for GPA on the Characteristic (B10.01) and Harmfulness (B10.02) dimensions. Behaviors are ordered in ascending order according to their mean Characteristic scores. Background colors in the Characteristic column indicate increasing levels of perceived typicality, ranging from light yellow for lower values to dark red for higher values (4.70 and above), with intermediate color shades representing successive score intervals.

### 3.8 Difficulties among former members

In Section B.11, participants were asked to indicate the extent to which they considered a range of post-exit difficulties as characteristic of former members of abusive groups, as well as the frequency and pervasiveness with which such difficulties are typically experienced. The results are presented in Table 5.

**TABLE 5:** Mean ratings of psychosocial difficulties among former members.

Domain	Characteristic M (SD)	Frequency/pervasiveness M (SD)
Low self-esteem	4.35 (0.82)	4.11 (0.91)
Shame and guilt	4.31 (0.89)	4.12 (0.88)
Anxiety and fear	4.30 (0.79)	4.14 (0.86)
Difficulties in adaptation and social integration	4.30 (0.77)	4.03 (0.91)
Identity crisis	4.21 (0.85)	3.95 (1.01)
Rumination on unpleasant memories and avoidance	4.18 (0.88)	4.04 (0.88)
Relational problems and loneliness	4.17 (0.80)	4.03 (0.86)
Feelings of grief and loss	4.16 (0.80)	3.92 (0.99)
Addictive behaviors	4.08 (0.94)	3.88 (0.92)
Sadness and despair	4.02 (0.86)	3.85 (0.93)
Impairments in decision-making	4.00 (0.91)	3.80 (0.94)
Paranoid and irrational ideation	3.90 (0.99)	3.81 (1.01)
Somatization	3.81 (0.95)	3.57 (1.01)
Anger and rage	3.77 (1.02)	3.77 (0.93)
Sleep disturbances	3.71 (1.02)	3.60 (0.99)
Dissociative states	3.60 (1.03)	3.58 (1.11)
Lack of social skills	3.60 (1.04)	3.63 (1.05)
Cognitive dullness	3.40 (1.11)	3.33 (1.15)
Sexual disorders	3.36 (1.04)	3.24 (1.07)
Eating disorders	3.10 (1.01)	3.13 (1.06)

Mean (M) and standard deviation (SD) of participants’ ratings of psychosocial difficulties among former members. Domains are ordered in ascending order according to their mean Characteristic scores. Background colors in the Frequency/Pervasiveness column indicate increasing levels of frequency and pervasiveness with which such difficulties are typically experienced, ranging from light yellow for lower values to dark red for higher values, with intermediate color shades representing successive score intervals.

### 3.9 Direct professional experiences and clinical profiles

In Section C of the questionnaire the data indicates that the majority of participants (57%) reported never having encountered abusive group-related issues within their professional practice. However, 43% of respondents reported having observed at least one critical episode, with the highest prevalence in the clinical sector (65%), followed by the private/corporate sector (17%), the forensic field (6%), other professional domains (7%), and the educational/school context (5%). A particularly noteworthy finding concerns the percentage of professionals who had personally managed situations linked to abusive groups: 47% of respondents reported having played an active role in the intervention. Only participants who provided a positive response were invited to answer the subsequent open-ended question regarding the most frequently reported diagnoses.

A total of 38 responses were provided by participants; of these, 36 were deemed eligible for analysis, as two responses contained content not pertinent to the question. Analysis of the most frequently reported diagnoses, based on open-ended responses, revealed a heterogeneous clinical picture, though with some recurring conditions^1^. Depression emerged as the most frequently mentioned diagnosis (*n* = 13; 36%), followed by anxiety disorders (*n* = 10; 28%). Personality Disorders – Cluster C were reported in nine cases (25%), of which dependent personality disorder was specifically mentioned in seven responses (19%). Dissociative disorders were identified in five cases (14%), as were post-traumatic stress disorder (14%) and Substance-Related and Addictive Disorders (14%). Personality Disorders – Cluster A accounted for four responses (11%), and dissociative identity disorder was also mentioned in four cases (11%). Personality Disorders – Cluster B were reported in three cases (8%), whereas obsessive–compulsive disorder was indicated in two cases (6%). Psychotic Spectrum Disorders were mentioned once (3%). Finally, nine responses (25%) referred to non-specific or unspecified diagnostic categories.

### 3.10 Appropriateness of terminology, psychological intervention and diagnostic instruments

In Section D, participants were presented with a series of expressions commonly used to describe abusive dynamics and were asked to rate their appropriateness on a 5-point Likert scale (1 = not at all, 5 = very much). Results are reported in Table 6.

**TABLE 6:** Perceived appropriateness of expressions referring to abusive mechanisms.

Expression	Mean (SD)
Psychological manipulation	4.79 (0.55)
Psychological/emotional abuse	4.67 (0.60)
Mind control	4.56 (0.75)
Psychological influence	4.54 (0.78)
Unethical manipulation	4.36 (0.96)
Thought reform	4.33 (0.91)
Spiritual abuse	4.21 (0.98)
Brainwashing	4.20 (1.11)
Induced conversion	4.12 (0.98)
Personality control	4.02 (1.05)

Regarding Section D2, participants were asked to rate the extent to which they considered it acceptable to administer psychological treatment interventions to members of abusive groups who do not explicitly seek such assistance. The mean score was *M* = 3.10 (SD = 1.27), indicating an overall moderate level of acceptability, accompanied by considerable variability among respondents. As shown by the frequency distribution, 14% of participants considered such interventions not acceptable, and 22% rated them as slightly acceptable. Approximately 19% expressed a neutral position, whereas 33% deemed these interventions acceptable and 13% regarded them as highly acceptable.

In Section D3, participants were also asked to indicate which diagnostic or assessment instruments they considered most suitable for investigating psychological functioning and vulnerability in GPA contexts. A total of 178 responses were provided by participants. Of these, 86% of participants (*n* = 153) reported no prior knowledge of the tools while 10 answer responses contained content not pertinent to the question; the remaining responses were included in the analysis. From the analysis a heterogeneous picture emerged regarding the instruments considered suitable for assessing psychological and behavioral aspects related to abusive group involvement. Validated instruments available in Italian included:

- Minnesota Multiphasic Personality Inventory-2 (*n* = 2; MMPI-2 Butcher, 2010),
- Millon Clinical Multiaxial Inventory (*n* = 1; MCMI-III/IV; Millon, 1987; Millon et al., 2015),
- Structured Clinical Interview for DSM-5 Personality Disorders (*n* = 1; SCID-5-PD; First et al., 2016),
- Rorschach Test (*n* = 1; Rorschach, 1921),
- Thematic Apperception Test (*n* = 2; TAT; Murray, 1943),
- Eysenck Personality Questionnaire-Revised (*n* = 1; EPQ-R; Eysenck and Eysenck, 1984),

Instruments mentioned but not validated in Italian included:

- Group Psychological Abuse Scale (*n* = 3; GPA; Chambers et al., 1994),
- Psychological Abuse Experienced in Groups Scale (*n* = 1; PAEGS; Saldaña et al., 2017),
- Behavior, Information, Thought, and Emotional control (BITE) Model (*n* = 1; Hassan, 2020),
- Central Relationship Questionnaire (*n* = 1; CRQ; Barber et al., 1998),
- Identity Style Inventory-5 (*n* = 1; ISI-5; Berzonsky et al., 2013),
- Multidimensional Locus of Control (*n* = 1; MLOC; O’Hea et al., 2009),
- Scala di Vulnerabilità a Gruppi Settari (*n* = 1; SGS).
- Revised Religious Fundamentalism Scale (*n* = 1; RRFS; Altemeyer and Hunsberger, 2004).

Several participants also referred to more general personality or psychosocial instruments, such as Big Five Inventory (*n* = 1; BFI; John et al., 1991), along with suggestions to integrate personality assessment tools with measures of social functioning.

## 4 Discussion

### 4.1 Overview

The present study sought to provide the first systematic examination of Italian psychologists’ awareness, representations, and professional experiences related to GPA, an area for which no epidemiological monitoring, institutional reporting, or professional benchmarks are currently available in Italy or in Europe. Against this background of conceptual ambiguity and data scarcity, understanding how psychologists perceive and interpret GPA is essential, given their central role in recognizing undue influence, supporting affected individuals, and informing interdisciplinary responses. By offering the first empirical snapshot of psychologists’ knowledge, terminology, informational sources, and perceived training needs, this study contributes foundational evidence to a field that remains largely unexplored in the Italian context.

### 4.2 Low formal knowledge but high perceived relevance

A consistent pattern emerging from the data is the coexistence of limited self-reported knowledge of GPA with a strong perception of its relevance and demand for specific training. Rather than indicating indifference or skepticism, this “knowledge–interest mismatch” points to a structural capacity gap within the profession. Several factors may help explain this pattern. First, psychologists may underestimate the prevalence or societal relevance of abusive groups due to the lack of epidemiological data and the low visibility of the phenomenon in academic and institutional discourse. Second, GPA is largely absent from Italian undergraduate and postgraduate psychology curricula, leaving most professionals without exposure to definitions, behavioral markers, or assessment frameworks. Third, the terminology surrounding GPA remains highly fragmented, with public and media discourse often relying on inconsistent or outdated labels (e.g., *sect*, *brainwashing*), which may contribute to confusion and discourage deeper engagement.

### 4.3 Reliance on non-academic information sources

A notable finding is that psychologists primarily rely on non-academic sources–particularly the internet and media–to obtain information about group psychological abuse. This pattern likely reflects a combination of structural factors: the limited availability of formal training pathways and professional guidelines in this domain, alongside the greater accessibility and visibility of online and media content compared to specialized scientific literature. Similar reliance on informal sources is common in under-institutionalized areas of practice, where authoritative frameworks and consolidated knowledge bases are lacking (Lilienfeld et al., 2012). However, this informational ecology introduces specific risks. Media and online narratives often prioritize sensational or extreme cases, reinforcing narrow and stereotyped representations of “sects” while overlooking the broader spectrum of psychologically abusive groups operating in non-religious or less conspicuous contexts (Robbins and Zablocki, 2001). Variability in source quality may therefore contribute to conceptual ambiguity and inconsistent definitions, potentially hindering clinicians’ capacity to distinguish coercive group dynamics from unconventional yet non-abusive forms of collective organization. Strengthening access to evidence-based terminology, empirically grounded frameworks, and structured professional training is thus essential to support accurate case conceptualization and competent practice in this area.

#### 4.3.1 Representations of groups

Psychologists primarily described abusive groups (both via open questions and structured ratings) in terms of psychological manipulation and control practices, and almost as many highlighted isolation and relational closure. This pattern closely matches contemporary psychosocial accounts of group psychological abuse, which define abusive groups primarily through observable influence and control processes – for example, behavioral, cognitive, emotional and relational restrictions (Hassan, 2020; Rodríguez-Carballeira et al., 2015). Importantly, when participants described group strategies more explicitly, they highlighted not only manipulation and isolation but also indoctrination, love bombing, threat, guilt induction, and progressive recruitment tactics. This constellation reflects mechanisms widely discussed in influence research, including incremental commitment, affective bonding, informational control, and identity reinforcement (Cialdini, 2009; Lalich, 2004). The co-occurrence of belonging-enhancing practices and abusive tactics suggests that psychologists recognize both the affiliative and restrictive components of high-control environments.

At the same time, structural characteristics such as strong leadership figures, dogmatic or absolute belief systems, hierarchical organization, exclusivity, rigid thinking, and totalizing dynamics, while recognized as representative of GPA contexts in closed-ended questions, were mentioned less frequently in responses to open-ended questions. This may indicate that practitioners intuitively prioritize overt relational strategies over systemic or ideological architecture. Yet contemporary scholarship emphasizes that undue influence is sustained not only through interpersonal manipulation but through the interaction between leadership structure, cognitive closure, and social containment (Lalich, 2004; Rodríguez-Carballeira et al., 2015). This shift toward a behaviorally anchored representation risks partially obscuring the ideological and authority-based mechanisms that sustain long-term compliance and identity restructuring within high-control systems.

#### 4.3.2 Representation of members

In contrast to the relatively process-oriented descriptions of abusive groups, psychologists’ representations of members were predominantly centered on individual vulnerability. Nearly two-thirds of respondents described members as fragile or psychologically vulnerable, suggesting that when attention shifts from group dynamics to individuals, explanatory emphasis tends to move from structural mechanisms to presumed personal predispositions. Notably, this representation was also the most strongly endorsed dimension in the Likert-scale ratings, followed closely by “suggestibility.” Part of this portrayal aligns with empirical findings indicating that transitional life phases and psychosocial stressors may temporarily increase openness to high-control environments (Aronoff et al., 2000; Levine and Salter, 1976). Similarly, references to loneliness and marginalization reflect documented recruitment pathways that leverage unmet affiliative needs and social isolation (Rodríguez-Carballeira et al., 2015; Singer and Lalich, 1995). Contemporary models, however, conceptualize susceptibility to undue influence as emerging from the interaction between situational stressors, relational dynamics, and systematic influence strategies, rather than from enduring personality profiles (Lalich and McLaren, 2010; Rodríguez-Carballeira et al., 2015). A minority of respondents attributed involvement to low education or limited critical thinking – a pattern mirrored in the structured ratings, receiving lowest endorsement. Such deficit-based explanations are not supported by available evidence, which indicates that individuals across educational and socioeconomic backgrounds may become involved, particularly during periods of identity transition or existential searching (Aronoff et al., 2000; Lalich, 2004).

Notably, almost half of respondents reported having directly managed cases involving individuals associated with psychologically abusive groups. The clinical presentations described were heterogeneous but showed recurrent internalizing patterns, particularly depressive and anxiety-related symptomatology. This is consistent with research indicating that prolonged exposure to coercive or controlling relational environments is associated with affective distress and stress-related outcomes (Aronoff et al., 2000; Lalich and McLaren, 2010). Personality-related formulations, especially Cluster C features such as dependency, were also mentioned. Yet empirical evidence does not support a deterministic personality-based vulnerability model (Aronoff et al., 2000). Such clinical impressions may instead reflect adaptive relational strategies within high-control systems or post-involvement meaning-making processes rather than stable predisposing traits. Finally, trauma-related and dissociative presentations were reported, aligning with trauma-informed frameworks that emphasize the cumulative psychological impact of emotional control, relational captivity, and prolonged social isolation (Cloitre et al., 2013; Herman, 2015). Consistent with these clinical impressions, Likert-scale evaluations indicated that participants considered affective dysregulation, identity confusion, relational difficulties, and trauma-related symptoms to be both characteristic and relatively pervasive among former members.

#### 4.3.3 Representations of leaders

Participants predominantly described leaders in terms of manipulation, charisma, and narcissistic grandiosity. This configuration closely aligns with contemporary models of high-control leadership in abusive groups, where charismatic authority is intertwined with strategic influence and relational dominance (Hassan, 2015; Lalich, 2004). Rather than depicting leaders merely as inspirational figures, respondents frequently framed them as exploitative and psychologically controlling, suggesting an awareness of the instrumental architecture underlying undue influence.

The recurrent pairing of charisma and narcissistic features is theoretically coherent. Research on destructive leadership indicates that charismatic appeal can function as a vehicle for self-enhancement and dependency induction when embedded in grandiose or entitled orientations (Conger, 1990; Padilla et al., 2007). In coercive group contexts, such dynamics may facilitate idealization processes and stabilize asymmetrical power relations.

Although some responses invoked antisocial or psychopathic traits, these were not central. This is noteworthy, as contemporary scholarship cautions against reducing abusive group dynamics to individual psychopathology alone. Instead, destructive leadership is increasingly conceptualized as emerging from the interaction between leader dispositions, follower needs, and structurally enabling environments (Padilla et al., 2007).

Overall, psychologists’ representations of leaders appear comparatively process-sensitive: manipulation, dominance, and narcissistic self-enhancement are recognized as core mechanisms of control. This contrasts with the more vulnerability-centered framing observed in representations of members, highlighting a subtle asymmetry in how agency and responsibility are distributed in professional narratives.

### 4.4 Terminology, assessment instruments, and ethical considerations

The frequent use of outdated or conceptually imprecise labels–such as *brainwashing* or *thought reform*–suggests that psychologists often rely on terminology rooted in historical or media narratives rather than contemporary scientific frameworks. These terms lack operational clarity, are tied to Cold War-era discourse, and are largely unsupported by empirical research (Lilienfeld et al., 2012). Their continued use risks reinforcing misconceptions and obscuring the dynamics captured by more precise constructs such as *group psychological abuse*, *abusive strategies*, and *undue influence*.

A similar pattern emerges in the domain of assessment and intervention. Participants predominantly reported using generic clinical tools–such as personality inventories or anxiety–depression measures–which, while useful for understanding individual distress, are not designed to assess undue influence or identity disruption within abusive groups. This reliance likely reflects the absence of validated Italian instruments capable of capturing group-specific dynamics. The limited adoption of tools informed by GPA research underscores the need for translations, cultural adaptations, and dissemination of validated measures.

Ethical considerations also surfaced in psychologists’ reluctance to intervene without explicit client consent. This hesitation aligns with core professional principles of autonomy and voluntary participation, and with legal constraints surrounding involuntary treatment. In cases involving undue influence, however, ethical practice requires balancing respect for autonomy with safeguarding concerns, particularly when individuals may be hesitant to seek help due to fear, loyalty, or manipulation.

### 4.5 Professional experiences

Only a minority of psychologists reported direct clinical or consultative experience with cases involving abusive groups. This limited exposure likely reflects the absence of institutional reporting systems, the covert nature of abusive group practices, and the broader lack of epidemiological or public health monitoring in Italy. It may also stem from the *reluctance or difficulty that current and former members face in seeking professional help*, a pattern frequently noted in the literature due to fear of retaliation, loyalty to the group, stigma, and uncertainty about whether clinicians will understand their experience (Lalich and Tobias, 2006; Matthews and Salazar, 2014). As a result, psychologists may encounter GPA-related distress without recognizing its origins or may struggle to contextualize clients’ symptoms within an abusive group environment.

Participants also described uncertainty regarding referral pathways, interprofessional collaboration, and the availability of specialized services. This mirrors findings in other areas of undue control, where professionals often report difficulties navigating cases that fall between mental health, social services, and legal frameworks (Stark, 2007).

Overall, the pattern suggests a field where perceived relevance is high, but professional experience is fragmented and inconsistently supported.

### 4.6 Training recommendations

The combined findings of limited formal knowledge, reliance on non-academic sources, conceptual ambiguity, and fragmented professional experience indicate a pressing need for structured, evidence-informed training on GPA. A key starting point is the development of shared professional language. Although no consensus-based terminology currently exists in the field, training can help consolidate clearer and more operational definitions by distinguishing undue influence from non-abusive group dynamics and by promoting the use of constructs supported by contemporary research over historically loaded or imprecise terms. Training should also enhance psychologists’ ability to formulate cases using relational, contextual, and socio-psychological mechanisms rather than relying solely on individual psychopathology.

In terms of delivery, accredited Continuing Professional Development (CPD) pathways are essential and should be supported by national and regional psychology boards to ensure quality and accessibility. Integrating GPA content into university curricula–particularly within clinical, forensic, and social psychology tracks–would address current educational gaps.

Finally, improving national infrastructure is critical. Establishing a specialized consultation network connecting clinicians with experts in GPA, forensic practice, and victim support services would provide essential guidance. Parallel efforts to adapt and validate GPA-specific assessment tools for the Italian context would strengthen diagnostic accuracy and facilitate coherent practice across settings.

### 4.7 Policy implications

The findings point to the need for stronger institutional support in addressing GPA as a public health and safeguarding concern. National and regional psychology boards could play a central role by recognizing GPA within professional development frameworks and promoting accredited training initiatives. At a broader level, the absence of epidemiological monitoring and coordinated referral pathways suggests the value of interinstitutional collaboration between mental health services, victim-support organizations, and legal actors. Joint training initiatives with forensic professionals could improve recognition and documentation of abusive group practices, while reducing reliance on inconsistent, individualized responses. Such measures would help situate GPA within a clearer professional and policy landscape and better support individuals affected by abusive environments.

### 4.8 Limitations and future directions

Some limitations should be considered when interpreting the present findings. The use of a convenience, self-selected sample introduces potential selection and response biases, as psychologists with prior interest or experience in the topic may have been more likely to participate than those with limited familiarity. Despite wide dissemination efforts, the overall participation rate was lower than expected, which may reflect either an underestimation of the phenomenon or a limited perception of its professional relevance. The heterogeneity of reported professional experiences further suggests that direct exposure to GPA cases is relatively uncommon, possibly due to gaps in training or the absence of structured educational opportunities. Finally, because responses to questionnaire items were not mandatory, some data were missing across specific questions, which should be considered when interpreting item-level results.

Future research should expand upon these preliminary findings by recruiting larger and more diverse samples across different regions and professional settings, ideally through more systematic or probability-based sampling strategies. Raising awareness of the topic within professional communities may also support higher participation rates in future studies. In methodological terms, further work is needed to adapt and validate specific assessment tools for the Italian context to ensure more accurate and comparable measurements. Finally, the substantial interest in training reported by participants highlights the importance of developing dedicated educational programs aimed at strengthening professionals’ competencies in recognizing and managing cases of group psychological abuse.

## 5 Conclusion

This study offers the first systematic overview of Italian psychologists’ awareness, representations, and professional experiences concerning group psychological abuse, addressing a longstanding gap in both national and international research. The findings reveal a clear discrepancy between low formal knowledge and high perceived relevance, a pattern consistent with the scarcity of training, institutional guidance, and epidemiological monitoring described in the literature. At the same time, participants’ representations of abusive groups, members, and leaders partially align with contemporary models of undue influence, while also diverging in ways that reflect culturally dominant narratives and terminological fragmentation. These dynamics have direct implications for practice, suggesting that psychologists recognize the importance of the phenomenon but lack the conceptual and structural resources needed to respond effectively.

Taken together, the results underscore the urgent need to support the profession through structured training, the development and dissemination of clearer shared terminology, and the establishment of interprofessional infrastructures capable of guiding assessment, referral, and collaboration across mental health, social, and legal systems. Strengthening these elements is essential for enhancing the capacity of psychologists to identify and address GPA, and for promoting a more coherent and evidence-informed response to an understudied yet clinically and socially significant phenomenon.

## Data Availability

The original contributions presented in this study are included in the article/Supplementary material, further inquiries can be directed to the corresponding author.

## Footnotes

1 It should be noted that the majority of responses referred to symptom presentations rather than to formal psychiatric diagnoses. For this reason, categories were constructed according to the diagnostic taxonomy of the last edition of the *Diagnostic and Statistical Manual of Mental Disorders* (DSM-5-TR, American Psychiatric Association, 2022). This approach allowed us to classify responses that mentioned specific disorders as well as those describing symptom clusters or clinical features that align with DSM-5-TR categories, ensuring both conceptual precision and inclusiveness of non-diagnostic descriptions.
