# Supplementary material for: Are psychologists aware of group psychological abuse? A survey on awareness of the phenomenon among Italian psychologists
Source: Front Psychol. 2026 Mar 26;17:1749074. doi: 10.3389/fpsyg.2026.1749074 (PMC13061703; doi:10.3389/fpsyg.2026.1749074)
Supplement: Supplementary file 1 [file Table_1.docx]

## **Supplementary Material**

### **Full Questionnaire: Items and Response Options**

### **Section A — Sociodemographic and Professional Profile**

1. Are you registered with the Order of Psychologists?

☐ Yes

☐ No

1. If yes, please specify the region of registration: __________
2. Register section:

☐ Section A

☐ Section B

1. Year of registration with the Order of Psychologists: __________
2. Year of birth: ________
3. Highest educational qualification obtained

☐ Bachelor’s degree

☐ Master’s degree

☐ Doctoral degree (PhD)

☐ Specialization in Psychotherapy

1. Title of Bachelor’s degree: __________
2. Title of Master’s degree: __________
3. Title of the doctoral degree: __________
4. School or area of specialization: __________
5. Have you also obtained one or more first- or second-level Master’s degrees?

☐ Yes

☐ No

1. Type of Master’s degree:

☐ First-level Master’s

☐ Second-level Master’s

1. Current professional field

☐ Clinical

☐ Organizational/private

☐ Forensic/legal

☐ School/educational

☐ Social intervention

☐ Other (please specify): __________

1. Years of professional experience:

☐ Less than 5 years

☐ 5–10 years

☐ 10–20 years

☐ More than 20 years

### **Section B1 — Self-Assessed Knowledge**

### In this section, you will be asked some general questions regarding your knowledge of the phenomenon of cults.

Please indicate the overall level of knowledge you think you have about cults.
*(1 = none, 5 = extensive)*

### **Section B2–B3 — Sources of Information and Perceived Quality**

Please indicate the amount of information you have received about cults through the following sources:
*(1 = none, 5 = extensive)*

- Scientific literature
- Popular magazines
- Newspapers or television
- Internet
- Clients you have worked with
- Training courses
- Seminars
- Professional colleagues
- Personal experiences

In general, I consider the quality of the information I have received about cults to be:
*(1 = very poor, 5 = excellent)*

### **Section B4 — Descriptions of Sectarian Groups (Open-Ended)**

Briefly describe the three main characteristics of sectarian groups.

*(Open-ended):* _______________________________________

### **Section B5 — Evaluative Labels for Sectarian Groups**

To what extent do the following statements describe cultic groups?
*(1 = not at all, 5 = completely)*

- Manipulative and violent groups
- Legitimate alternative groups
- Minority and divergent groups
- Radical and extremist groups
- Deviant religious groups
- Exploitative groups

### **Section B6 — Descriptions of Members (Open-Ended)**

Briefly describe the three main characteristics of people who join a cult.

*(Open-ended):* _______________________________________

### **Section B7 — Characteristics of Members**

To what extent do the following characteristics describe people involved in a cult?
*(1 = not at all, 5 = completely)*

- Prior psychological disorders
- Weakness of character
- Low level of education
- Emotional vulnerability
- Susceptibility to influence

### **Section B8 — Descriptions of Leaders (Open-Ended)**

Briefly describe the three main characteristics that, from your point of view, define the leaders of a cult.

*(Open-ended): _*______________________________________

### **Section B9 — Group Strategies (Open-Ended)**

Briefly describe the three most commonly used strategies in cultic groups.

*(Open-ended): __*_____________________________________

### **Section B10 — Group Behaviors and Perceived Harmfulness**

You will find below a list of behaviors that may occur within groups.

Please indicate:

(a) how characteristic you consider these behaviors of sectarian groups, and
*(1 = not characteristic, 5 = completely characteristic)*

(b) to what extent you think they could harm group members.
*(1 = not at all, 5 = extremely)*

**Behaviors:**

- Requiring members to fully identify with the group ideology
- Granting absolute authority to the leader
- Monitoring members’ behavior
- Controlling members’ finances
- Controlling members’ activities and use of time
- Psychophysical weakening of members
- Denigrating, humiliating, or rejecting group members
- Inducing guilt and negatively reconstructing members’ past identity
- Manipulating language by altering the meaning of words
- Attacking critics through ad hominem arguments
- Idealizing the group and rejecting everything outside the group

### **Section B11 — Difficulties Among Former Members**

Below is a list of psychosocial difficulties.

Please indicate:

(a) how common these difficulties are among former cult members, and

(b) how pervasive and persistent they are.

*(1 = not at all, 5 = very much)*

**Difficulties:**

- Relational problems and loneliness
- Somatization
- Paranoid and irrational ideation
- Identity crisis
- Sadness and despair
- Difficulties in social integration and adjustment
- Addictive behaviors
- Cognitive rigidity
- Lack of social skills
- Decision-making difficulties
- Eating behavior disturbances
- Dissociative states
- Feelings of shame and guilt
- Rumination on unpleasant memories and avoidance
- Sexual dysfunctions
- Sleep disturbances
- Low self-esteem
- Feelings of pain and loss
- Anxiety and fear
- Anger and rage

### **Section C1 — Direct Professional Experiences**

In this section, we will ask you about your professional experience in this type of situation.

In your professional experience (regardless of the field in which you specialized or currently work), have you encountered at least one problem related to cults?

☐ Yes

☐ No

The field of work in which I encountered the highest number of cult-related cases was:

☐ Clinical

☐ Organizational/private

☐ Forensic/legal

☐ School/educational

☐ Other (please specify): __________

Have you personally had the opportunity to intervene in a situation related to a cult-related problem?

☐ Yes

☐ No

In such cases, what were the most frequently recognized diagnoses?

*(Open-ended): __*_____________________________________

### **Section D1–D3 — Terminology and Diagnostic Instruments**

How appropriate do you think the following expressions are when referring to cults?
*(1 = not at all, 5 = very much)*

Expressions:

- Mind control
- Thought reform
- Brainwashing
- Psychological manipulation
- Unethical manipulation
- Psychological influence
- Induced conversion
- Personality control
- Psychological/emotional abuse
- Spiritual abuse

To what extent would you consider it acceptable to carry out psychological treatment interventions on cult members when they do not wish to receive them?

*(1 = completely unacceptable, 5 = completely acceptable)*

Do you know any specific psychometric tools for assessing individuals who are involved in a cult?

*(Open-ended):* _______________________________________

### **Section D4–D5 — Training Interest and Feedback Preferences**

Do you consider it important that the CeSAP association carries out training activities on this topic?

☐ Yes

☐ No

Would you be interested in knowing the results of this survey?

☐ Yes

☐ No
